# Gut microbiota steroid sexual dimorphism and its impact on gonadal steroids: influences of obesity and menopausal status

**DOI:** 10.1186/s40168-020-00913-x

**Published:** 2020-09-20

**Authors:** Jordi Mayneris-Perxachs, María Arnoriaga-Rodríguez, Diego Luque-Córdoba, Feliciano Priego-Capote, Vicente Pérez-Brocal, Andrés Moya, Aurelijus Burokas, Rafael Maldonado, José-Manuel Fernández-Real

**Affiliations:** 1 Department of Endocrinology, Diabetes and Nutrition, Departament de Ciències Mèdiques, Hospital of Girona “Dr JosepTrueta”, Girona Biomedical Research Institute (IdibGi), University of Girona, Carretera de França s/n, 17007 Girona, Spain; 2 CIBERobn Pathophysiology of Obesity and Nutrition, Instituto de Salud Carlos III, Madrid, Spain; 3 Maimónides Institute of Biomedical Research (IMIBIC), Reina Sofía University Hospital, University of Cordoba, Cordoba, Spain; 4 CIBERfes Frailty and Healthy Aging, Instituto de Salud Carlos III, Madrid, Spain; 5 Department of Genomics and Health, Foundation for the Promotion of Health and Biomedical Research of Valencia Region (FISABIO-Public Health), Valencia, Spain; 6 CIBER in Epidemiology and Public Health (CIBEResp), Madrid, Spain; 7 Institute for Integrative Systems Biology (I2SysBio), The University of Valencia and The Spanish National Research Council (CSIC-UVEG), Valencia, Spain; 8 Laboratory of Neuropharmacology, Department of Experimental and Health Sciences, Universitat Pompeu Fabra, Barcelona, Spain; 9 Present address: Institute of Biochemistry, Life Sciences Center, Vilnius University, Vilnius, Lithuania; 10 Hospital del Mar Medical Research Institute (IMIM), Barcelona, Spain

**Keywords:** Sex, Gender, Gonadal steroids, Testosterone, Progesterone, Microbiome, Sexual dimorphism

## Abstract

**Background:**

Gonadal steroid hormones have been suggested as the underlying mechanism responsible for the sexual dimorphism observed in metabolic diseases. Animal studies have also evidenced a causal role of the gut microbiome and metabolic health. However, the role of sexual dimorphism in the gut microbiota and the potential role of the microbiome in influencing sex steroid hormones and shaping sexually dimorphic susceptibility to disease have been largely overlooked. Although there is some evidence of sex-specific differences in the gut microbiota diversity, composition, and functionality, the results are inconsistent. Importantly, most of these studies have not taken into account the gonadal steroid status. Therefore, we investigated the gut microbiome composition and functionality in relation to sex, menopausal status, and circulating sex steroids.

**Results:**

No significant differences were found in alpha diversity indices among pre- and post-menopausal women and men, but beta diversity differed among groups. The gut microbiota from post-menopausal women was more similar to men than to pre-menopausal women. Metagenome functional analyses revealed no significant differences between post-menopausal women and men. Gonadal steroids were specifically associated with these differences. Hence, the gut microbiota of pre-menopausal women was more enriched in genes from the steroid biosynthesis and degradation pathways, with the former having the strongest fold change among all associated pathways. Microbial steroid pathways also had significant associations with the plasma levels of testosterone and progesterone. In addition, a specific microbiome signature was able to predict the circulating testosterone levels at baseline and after 1-year follow-up. In addition, this microbiome signature could be transmitted from humans to antibiotic-induced microbiome-depleted male mice, being able to predict donor’s testosterone levels 4 weeks later, implying that the microbiota profile of the recipient mouse was influenced by the donor’s gender. Finally, obesity eliminated most of the differences observed among non-obese pre-menopausal women, post-menopausal women, and men in the gut microbiota composition (Bray-Curtis and weighted unifrac beta diversity), functionality, and the gonadal steroid status.

**Conclusions:**

The present findings evidence clear differences in the gut microbial composition and functionality between men and women, which is eliminated by both menopausal and obesity status. We also reveal a tight link between the gut microbiota composition and the circulating levels of gonadal steroids, particularly testosterone.

Video Abstract

## Background

The gut microbiota composition is known to be changed in parallel to a myriad of environmental factors, being diet and antibiotic/drug exposures the main determinants. There is some evidence that sex may influence the diversity, composition, and function of gut bacterial microbiota, although the results are inconsistent. Initial studies evaluating a relatively small number of subjects with limited technical capabilities, showed little or no significant differences attributed to gender. In a 2005 study on 91 subjects of northern European origin (France, Denmark, Germany, the Netherlands, and the UK), there were no significant differences in gut microbiota between sexes according to principal component analysis [1]. A 2006 study, conducted in samples from France, Germany, Italy, and Sweden found higher levels of the *Bacteroides-Prevotella* group among males [2]. In a 2008 Chinese study using group-specific DGGE profiling of *Bacteroides* spp., a higher abundance of *Bacteroides thetaiotaomicron* was found among males [3]. More recent large population-wide studies also failed to show any major sex-specific differences in the gut microbiota diversity, complexity, or composition [4–6].

However, some other recent studies have highlighted the importance of sex as a factor affecting the human gut microbiota. Several studies have reported that men have lower microbial diversity than women [6–8]. In a large cohort study involving two independent extensively phenotyped cohorts, the Belgian Flemish Gut Flora Project (*n* = 1,106) and the Dutch LifeLines-DEEP study (*n* = 1,135), sex had a 10th effect size among 69 factors that were shown to correlate significantly with overall microbiome community variation [7]. In another study, sex was also associated with the gut microbiota composition overall, with women having lower abundance of Bacteroidetes [9]. Comparisons at the species level in a Dutch cohort study reported sex associations with twelve microbial species (including *Alistipes onderdonkii, Bilophila*, *Lachnospiraceae*, and *Bifidobacterium* species increased in women) [8]. After multiple corrections, the relative abundance of only *Akkermansia muciniphila* was higher in females. Analysis of a healthy cohort of 300 individuals revealed that men were three times more likely than women to harbor stool community type with higher relative abundances of *Ruminococcaceae*, *Faecalibacterium*, and *Alistipes*, but lower *Bacteroides* and lack of *Prevotella* [10]*.* In one Japanese study, significantly higher levels of *Prevotella*, *Megamonas*, *Fusobacterium*, and *Megasphaera* were found among males, while *Bifidobacterium*, *Ruminococcus*, and *Akkermansia* were higher among females [11]. *Ruminococcus* was also more abundant among Chinese females compared with males [12]. However, the results of each study regarding the differences in microbial taxa between sexes are inconsistent.

Of note, the majority of studies evaluating sex differences in gut microbiota did not take into account the influence of menopausal status. Menopausal status is known to affect gut microbiome, with premenopausal women having a higher abundance of SCFA producing bacteria compared to postmenopausal women and age-matched men [13, 14]. Specifically, the relative abundances of the SCFA producing genera *Prevotella*, *Ruminococcus*, and *Roseburia* were reported to depend both on sex and hormonal status. In a recent study involving three large cross-sectional cohorts, the association between sex and alpha diversity was much stronger in young adults than in middle-aged adults [15] and no differences were observed between men and women with a mean age of 60 years [16]. This suggests the menopause may play a role in the inconsistent results observed in all the previous studies.

Despite the role for female sex hormones in gastrointestinal transit [17, 18], clinical evidence for a change in gut microbiota composition according to circulating hormonal status is very limited in humans. Correlations with estradiol levels suggest positive associations with Gammaproteobacteria class and unknown family from *Mixococcales* (Proteobacteria-Lipopolysaccharide (LPS) producers), while bacterial family *Prevotellaceae* was negatively correlated [13]. Oral contraceptives and ovariectomy are also associated with changes in gut microbiota [8]. Importantly, castration in male mice abolishes sexual difference of gut microbiota composition, suggesting a critical role for testosterone in the complexity and diversity of virile males [19].

Given all these evidences, we hypothesized changes in gut microbiota composition and functionality according to sex and menopausal status. We also aimed to evaluate the potential influence of circulating gonadal steroids, as very few studies have studied these important confounding factors. We found that testosterone was the main factor associated with the gut microbiota signature. Finally, we explored whether this signature could be transmitted to mice using microbiota transplantation.

## Results

Clinical characteristics of the study subjects at baseline (*n* = 131) and after 1-year follow-up (*n* = 81) are described in Table 1 and Supplementary Table 1, respectively.

**Table 1:** Baseline characteristics of subjects according to the gender and menopausal status

**Clinical data**	**Pre-menopausal (*n*** **= 44)**	**Post-menopausal (*n*** **= 45)**	**Men (*n*** **= 42)**	***P***
Age	41.9 [34.4-48.4]	58.6 [55.4-59.6]	46.1 [39.2-54.3]	< 0.001
Polycystic ovary syndrome (%)	3 (7%)	0 (0%)	0 (0%)	0.05
Alcohol intake (g/day)	0.0 [0.0-2.1]	0.86 [0.0-2.5]	2.3 [0.0-12.3]	0.003
Smoking (no, former, yes) (%)	63.6, 27.3, 9.1	46.7, 44.4, 8.9	51.9, 35.9, 12.2	0.202
Obesity (%)	61.4	55.6	57.1	0.849
BMI (kg/m^2^)	39.5 [25.0-44.3]	32.8 [24.4-41.4]	34.1 [27.5-45.1]	0.304
Fat mass (kg)	46.3 [21.8-61.3]	39.8 [22.6-50.3]	34.5 [23.5-61.1]	0.530
SBP (mmHg)	128.5 (18.6)	131.0 (18.7)	133.5 (20.4)	0.088
DBP (mmHg)	73.5 (12.8)	76.5 (9.3)	76.0 (11.8)	0.276
HDL cholesterol (mg/dL)	52.0 [45.5-62.0]	62.0 [51.5-72.5]	48.5 [37.8-58.0]	< 0.001
Triglycerides (mg/dL)	79.5 [58.7-106.7]	96.0 [72.5-132.0]	104 [67.5-162.7]	0.071
Fasting plasma glucose (mg/dl)	94.0 [88.0-98.8]	96.0 [90.5-104.5]	98.0 [90.8-104.0]	0.197
HOMA-IR	5.68 [2.56-8.02]	3.01 [1.78-5.90]	3.57 [2.35-6.44]	0.051
IVGTT (80-120 min) (mg/dL)	106.7 (8.18)	103.1 (8.69)	103.5 (9.16)	0.162
M-clamp (mg/(kg·min))	5.30 [2.50-10.4]	6.86 [4.60-10.3]	5.01 [2.61-8.39]	0.137
HbA1c (%)	5.50 (0.29)	5.56 (0.32)	5.52 (0.29)	0.606
hs-CRP (mg/dL)	3.24 [0.91-9.49]	2.53 [0.64-6.10]	1.69 [0.74-3.14]	0.065

Results are expressed as number and frequencies for categorical data, mean and standard deviation for normal distributed continuous variables, and median and interquartile range for non-normal distributed continuous variables

Differences between groups were assessed using the *χ*^2^test for categorical variables, one-way ANOVA for normal quantitative variables, and the Kruskal-Wallis test for non-normal quantitative variables

*BMI* body mass index, *DBP* diastolic blood pressure, *Hb1Ac* glycated hemoglobin, *HDL* high density lipoprotein, *hs-CRP* high sensitive C-reactive protein, *IVGTT* intravenous glucose tolerance test, *SBP* systolic blood pressure

### Influences of gender and menopausal status on the gut microbiome composition

**Fig. 1:**
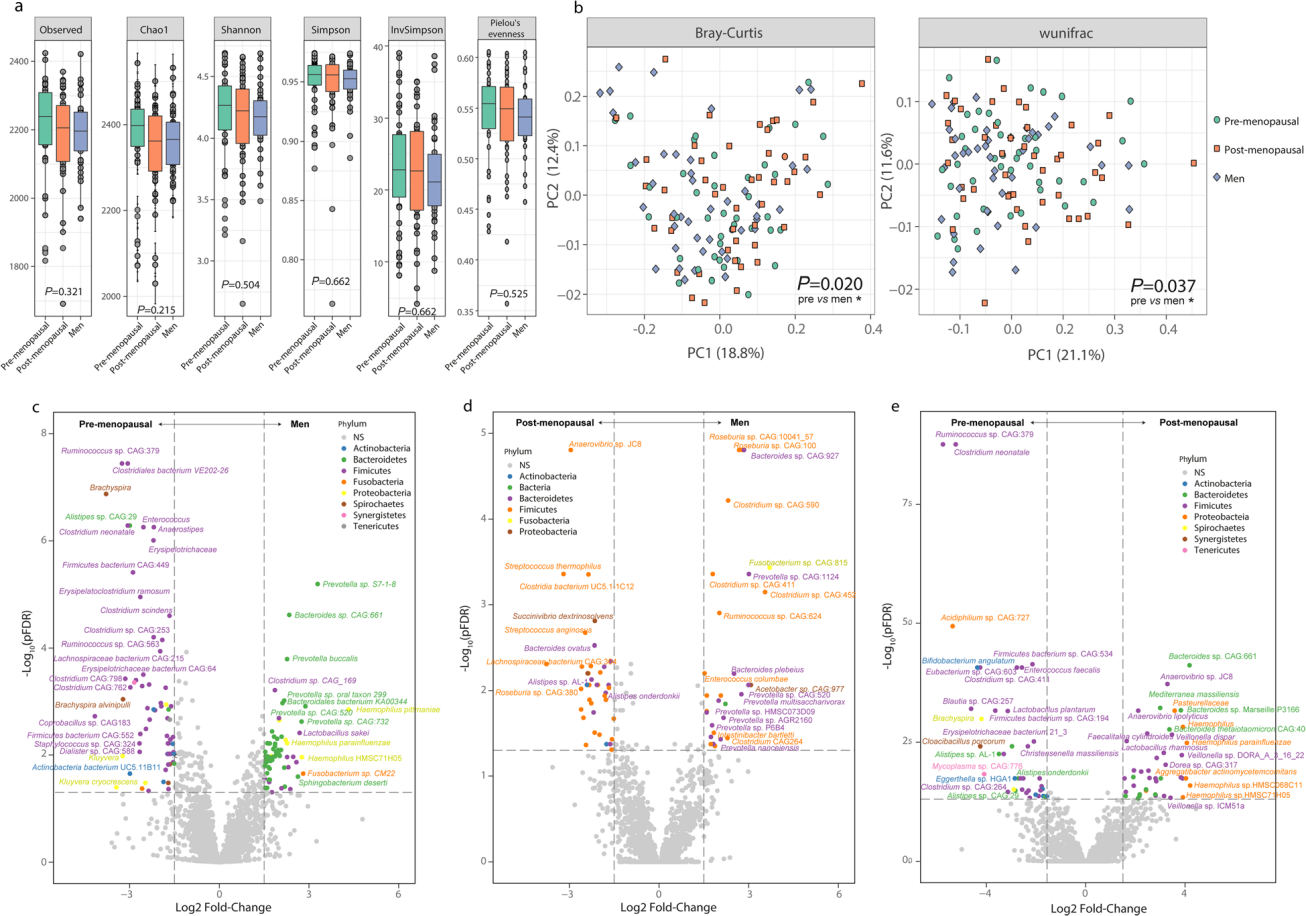
Associations of gut microbiota composition with gender and menopause status. (**a**) Alpha diversity indices (*n* = 131). (**b**) Beta diversity measured by Bray Curtis and weighted unifrac. Overall differences in the microbiome composition among groups were assessed by PERMANOVA using 1000 permutations and pairwise differences between groups were assessed using the pairwise.adonis function adjusted for Bonferroni correction. *p < 0.05. (**c**) Volcano plot of differential bacterial abundance analysis between pre-menopausal women and men, (**d**) post-menopausal women and men, and (**e**) pre- and post-menopausal women, as calculated by DESeq2 from shotgun metagenomic sequencing, adjusting for age and obesity status. For each taxa, the fold change and the *p* values corrected for multiple comparisons by the Benjamini-Hochberg procedure (*p*FDR) are plotted. Significantly different taxa (FC > 1.5 and *p*FDR < 0.05) are colored according to phylum

We did not find significant differences in alpha diversity indices among pre-menopausal women, post-menopausal women, and men (Fig. 1a). However, Bray-Curtis and weighted unifrac beta diversity measures both indicated significant differences in the microbiome composition among groups (Fig. 1b). In particular, pre-menopausal women had different microbial composition compared to men based on either Bray-Curtis (*P* = 0.042) or weighted unifrac (*P* = 0.044) beta diversity, whereas no differences were observed between the other groups. When subjects were studied according to the obesity status, no differences were found in any of the alpha diversity indices either in non-obese (Supplementary Figure 1a) or obese individuals (Supplementary Figure 1b), although in the former group, pre-menopausal tended to have higher alpha diversity than post-menopausal women and men based on the Chao1 index. Bray-Curtis and weighted unifrac beta diversity measures revealed different microbiome compositions between pre-menopausal women and men (*P* = 0.008 and *P* = 0.004, respectively) and post-menopausal women and men (*P* = 0.042 and *P* = 0.015, respectively) in non-obese subjects (Supplementary Figure 1c,d). Remarkably, these differences were eliminated in obese subjects (Supplementary Figure 1e,f).

Differential abundance taxa between pre-menopausal women, post-menopausal women, and men were identified using DESeq2 analysis adjusting for age and obesity. We identified 273 taxa with significantly different read counts between pre-menopausal women and men (Fig. 1c, Supplementary Table 2). Compared to pre-menopausal women, a lower number of taxa differed from men (103) in post-menopausal women (Fig. 1d, Supplementary Table 3). A total of 90 taxa were differentially abundant between pre-menopausal and post-menopausal women (Fig. 1e, Supplementary Table 4).

At the species level, men and post-menopausal women had higher abundances of several species belonging to the *Pasteurellaceae* (*Haemophilus*sp. HMSC71H05, *Haemophilus parainfluenzae*), Bacteroidaceae (*Bacteroides* sp. CAG:661, *Bacteroides stercoris* CAG:120, *Mediterranea massiliensis*, *Bacteroides pectinophilus* CAG:437), *Prevotellaceae* (*Prevotella* sp. P6B1, *Prevotella* sp. CAG:617), and *Clostridiaceae* families (*Clostridium* sp. CAG:169, butyrate-producing bacterium SS3/4), but lower abundances of several species, particularly *Ruminococcus*sp. CAG:379, *Clostridium neonatale*, *Alistipes*sp. CAG:29, *Cloacibacillus porcorum*, *Erysipelotrichaceae bacterium CAG:64*, and *Brachyspira*, compared to pre-menopausal women.

Compared to pre- and post-menopausal women, men had higher abundances of several *Prevotellaceae* species (*Prevotella* sp. CAG:520, *Prevotella multisaccharivorax*, *Prevotella* sp. AGR2160, *Prevotella nanceiensis*, *Prevotella* sp. HMSC073D09, *Prevotella bergensis*, *Prevotella* sp. CAG:924, *Prevotella ruminicola*) and *Clostridium* sp. CAG:277, but lower levels of *Roseburia *sp. CAG:380, *Firmicutes bacterium* CAG:884, *Lachnospiraceae bacterium* CAG:364, *Bacillus* sp. CAG:988, *Succinivibrio dextrinosolvens*, *Clostridium* sp. CAG:557, *Bacteroides dorei *CAG:222, and *Lachnospiraceae bacterium* CAG:215.

At the family level, men had higher *Pasteurellaceae*, *Cytophagaceae*, *Idiomarinaceae*, *Prevotellaceae*, *Sphingobacteriaceae*, *Flavobacteriaceae*, and *Fibrobacteriaceae*, whereas the abundance of *Synergistaceae*, *Fusobacteriaceae*, *Myxococcaceae*, *Actinobacteria*, *Enterococcaceae*, and *Christensenellaceae* was higher in pre-menopausal women (Supplementary Figure 1a). Once again, men had higher abundances of *Fibrobacteriaceae* and *Sphingobacteriaceae* compared to post-menopausal women, whereas the latter had higher levels of *Acetobacteriaceae*, *Leuconostocaceae*, *Acholeplasmataceae*, *Lactobacillaceae*, *Streptococcaceae*, and *Lentisphaerae* (Supplementary Figure 1b). Post-menopausal women had higher abundances of *Pasteurellaceae*, *Comamonadaceae*, and *Lentisphaerae*, but lower abundances of *Acholeplasmataceae*, *Enterococcaceae*, *Micrococcales*, and *Christensenellaceae* than pre-menopausal women (Supplementary Figure 1c).

### Influence of gender and menopausal status differences on the gut microbiome functionality

**Fig. 2:**
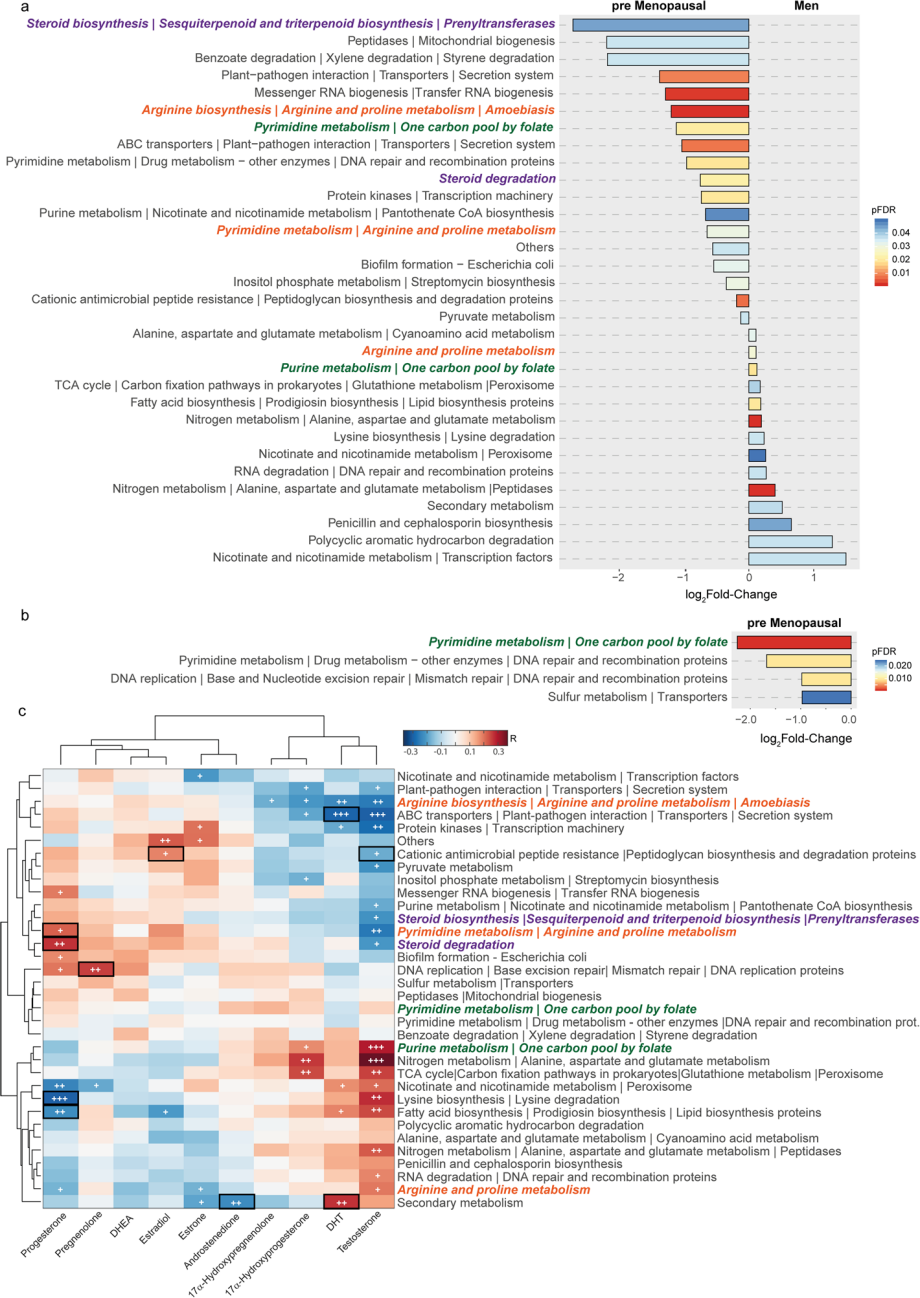
Associations of gut microbiota functionality with gender and menopause status. (**a**) Fold change for the significant differential KEGG pathways between pre-menopausal women and men, and (**b**) pre- and post-menopausal women, identified by DESeq2 adjusting for age and obesity status. Bars are colored according to the Benjamini-Hochberg corrected *p* values (*p*FDR). (**c**) Spearman correlation heatmap among the abundance of identified KEGG pathways and plasma concentrations of gonadal steroids. Clustering was based on Euclidean distances and Ward linkage. Significance: +, < 0.05; ++, < 0.01; +++,< 0.001. Significant associations after adjusting for age, obesity, and sex are highlighted with a black box

Metagenome functional analyses based on KEGG pathways revealed significant differences between premenopausal women and men (Fig. 2a). Remarkably, enrichment in steroid biosynthesis and degradation of metabolic pathways were found in the gut microbiota of premenopausal women compared to men. Other differentially expressed pathways included arginine biosynthesis and metabolism, purine and pyrimidine metabolism, one carbon pool by folate and carbohydrate metabolism pathways. Conversely, we did not find significant differences in the microbiome functionality between postmenopausal women and men, despite having different bacterial composition. Indeed, we only found an enrichment in arginine biosynthesis and metabolism pathways in postmenopausal women compared to men (log_2_Fold-change = −1.33, *p*FDR = 0.034), which was also increased in premenopausal women compared to men. In line with the functional differences between premenopausal women and men, the gut microbiota of premenopausal women was enriched in pyrimidine and one carbon pool by folate pathways compared to postmenopausal women (Fig. 2b). In addition, these differences were blunted by obesity. Therefore, while these functional differences between premenopausal women and men and between pre- and postmenopausal women were evident in non-obese individuals (Supplementary Figure 2a,b), no differences in the gut microbiota functionality were observed in obese subjects. Finally, most of the identified bacterial pathways had significant correlations with the plasma levels of gonadal steroids, particularly progesterone and testosterone (Fig. 2c). Interestingly, after adjustment for age, obesity, and menopause status, the steroid degradation pathway had a significant positive correlation with progesterone levels.

### Gender, menopausal status, and obesity differences in the gonadal steroids

**Fig. 3:**
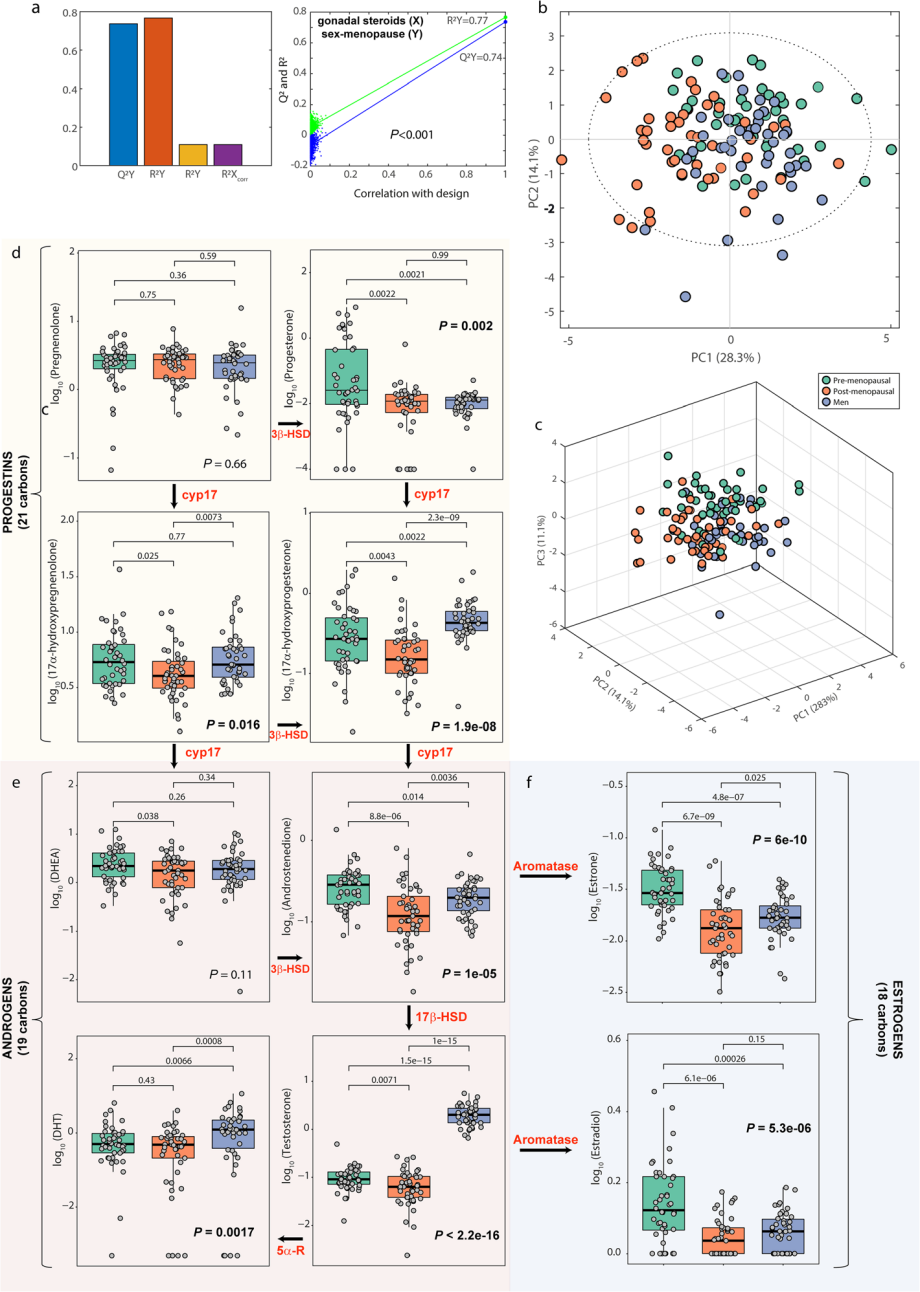
Gender and menopausal status differences in gonadal steroids. (**a**) Goodnessoffit (*R*^2^*Y*), goodness of prediction (*Q*^2^*Y*), and permutation tests for the O-PLS-DA model predicting the sex and menopause status from the circulating gonadal steroid levels. (**b**, **c**) Principal component analysis score plots for the plasma levels of gonadal steroids colored according to the gender group. (**d**) Boxplots for the concentrations of progestin, (**e**) androgens, and (**f**) estrogens converted to base 10 logarithmic values. Differences among groups were analyzed by a Kruskal-Wallis test, and pair-wise comparisons were assessed by Wilcoxon test. Significant differences are highlighted in bold

A significant O-PLS-DA model predicted the gender and menopause status from the levels of several steroids (Fig. 3a). Unsupervised analyses by principal component analysis also showed a clear between-group difference (Fig. 3b, c). Among steroids, estrone, estradiol, androstenedione, testosterone, DHT (Dihydrotestosterone), Progesterone, 17α-hydroxyprogesterone, and 17α-hydroxypregnenolone have significant between-group differences (Fig. 3d-f). As expected, males had higher levels of testosterone, DHT, and 17α-hydroxyprogesterone compared to both pre- and post-menopausal women. Pre-menopausal women had higher estrone, estradiol, progesterone, and androstenedione compared to men, whereas post-menopausal women had lower estrone, androstenedione, and 17α-hydroxypregnenolone compared to men. The levels of estrone, estradiol, androstenedione, testosterone, progesterone, 17α-hydroxypregnenolone, and 17α-hydroxyprogesterone were higher in pre-menopausal compared to post-menopausal women. Remarkably, most of these differences were lost in obese subjects (Supplementary Figure 3). In particular, while lean pre-menopausal women had higher circulating levels of testosterone, DHEA (dehydroepiandrosterone), progesterone, 17α-hydroxyprogesterone, and 17α-hydroxypregnenolone, compared to lean post-menopausal women, no significant differences were found in the levels of these steroids between obese pre- and post-menopausal women. In addition, estrogens levels were higher in obese post-menopausal women compared to lean post-menopausal women, whereas no differences were found between obese and lean pre-menopausal women. This is in agreement with the fact that pre-menopausal women mainly synthesize estrogens in the ovary, while in post-menopausal women ovarian biosynthesis is replaced by peripheral site synthesis, and in obese postmenopausal women, adipose tissue is the main source of estrogen biosynthesis.

### Gut microbial associations with gonadal steroids

**Fig. 4:**
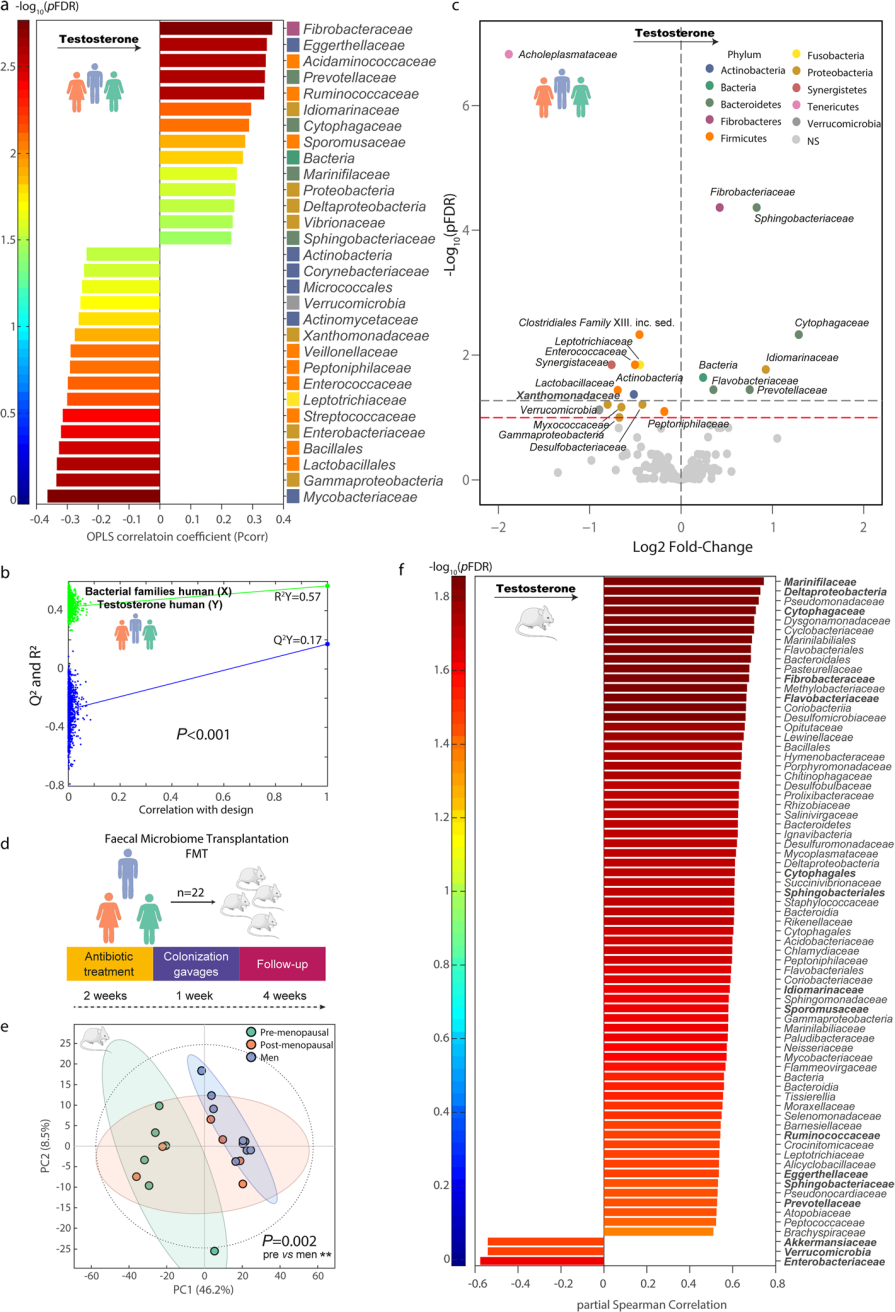
Gut microbial associations with circulating testosterone concentrations. (**a**) Significant gut bacterial families predicting plasma testosterone levels in humans identified by O-PLS modeling. (**b**) Permutation tests for the goodness-of-fit (*R*^2^*Y*) and goodness of prediction (*Q*^2^*Y*) for the O-PLS model between bacterial families and circulating testosterone concentrations in humans. (**c**) Volcano plot of gut bacterial families associated with testosterone levels identified by DESeq2, adjusting for age and obesity status. For each family, the fold change and the Benjamini-Hochberg corrected *p* values (*p*FDR) are plotted. Significant families (gray dashed line: *p*FDR < 0.05; red dashed line: *p*FDR < 0.1) are colored according to phylum. (**d**) Experimental design for the fecal microbiota transplantation study in mice. Fecal samples from 22 human donors (11 men and 11 women) were transplanted to 22 mice after 2 weeks of antibiotic treatment. After 28 days of colonization gavage, mice fecal samples were collected and analyzed by shotgun metagenomic sequencing. (**e**) Principal component analysis score plot based on recipient’s mice bacterial families colored according to human donor sex and menopause status. Overall differences in the microbiome composition were assessed by PERMANOVA using 1000 permutations and Euclidean distances. Pairwise differences between groups were assessed using the pairwise.adonis function adjusted for Bonferroni correction. **p < 0.01. (**f**) Significant recipient’s mice bacterial families predicting human donor circulating testosterone levels identified by partial Spearman correlation adjusted by age and obesity status

We then evaluated the possible associations between gut microbiota composition and the circulating concentrations of the main gonadal steroids. A significant O-PLS model was obtained for the prediction of testosterone (Fig. 4a, b) levels from microbiota families. In particular, *Prevotellaceae*, *Cytophagaceae*, *Fibrobacteriaceae*, *Sphingobacteriaceae*, and *Idiomarinaceae* were positively associated with testosterone levels, whereas several families from the Actinobacteria, Proteobacteria, Firmicutes, and Verrucomicrobia phylum were negatively associated with testosterone levels. We further evaluated the association between the gut microbiota families and the circulating testosterone levels using a negative binomial distribution by DESeq2 and adjusting for age and obesity (Fig. 4c). Again, *Fibrobacteriaceae*, *Idiomarinaceae*, and several families from the Bacteroidetes phylum (*Sphingobacteriaceae*, *Cytophagaceae*, *Prevotellaceae*, and *Flavobacteriaceae*) still had positive associations with testosterone levels, whereas *Acholeplasmataceae*, Verrucomicrobia, and several families from the Proteobacteria (*Gammaproteobacteria*, *Myxococcaeae*, *Xanthomonadacea*), Firmicutes (*Lactobacillaceae*) and Actinobacteria phyla had the strongest negative fold change. We were also able to predict the circulating progesterone levels from the gut microbiota composition, although the predictive performance of the O-PLS model was lower (*Q*^2^*Y* = 0.04, *P* = 0.003).

When we considered the obesity status, better predictive models for the testosterone (Supplementary Figure 4a,b) and progesterone (*Q*^2^*Y* = 0.11, *P =* 0.008) levels were obtained in non-obese individuals, whereas the gut microbiota composition of obese subjects could not predict the circulating concentrations of these two gonadal steroids (*Q*^2^*Y* = −0.12 and *Q*^2^*Y* = −0.25). Noteworthily, the same bacterial family signature was still positively associated with testosterone levels in non-obese subjects, whereas Verrucomicrobia and *Akkermansiaceae* (also from Verrucomicrobia phylum) were predictive of lower circulating testosterone concentrations.

The gut microbiota composition was also able to predict the plasma testosterone levels (*n* = 81) 1 year later (Supplementary Figure 4c). In particular, we identified a consistent microbial signature positively associated with testosterone concentrations that included *Cytophagaceae*, *Prevotellaceae*, *Fibrobacteriaceae*, *Sphingobacteriaceae*, *Flavobacteriaceae*, and *Idiomarinaceae* (Supplementary Figure 4d). Once again, several families from the Verrucomicrobia, including *Optitutaceae*, *Puniceicoccaceae*, and an unknown family of Verrucomicrobia, have negative associations with testosterone.

### The sex-related signature is transmitted to mice through the gut microbiota

We finally evaluated the hypothesis that the sex-related signature could be transplanted to mice through the gut microbiota. We transplanted the microbiota from 11 men and 11 women (age and obesity matched) to 22 male mice (Fig. 4d). Fecal microbiota transplantation from human donor to recipient mice resulted in a clear difference in the microbiome composition of male mice that received microbiota from pre-menopausal women and those that transplanted with microbiota from male donors 28 days after transplantation (*P* = 0.006) (Fig. 4e). Remarkably, most mice receiving microbiota from obese post-menopausal women had a microbiota profile similar to those that received microbiota from male donors (Fig. 4e).

Interestingly, when evaluating the mice gut microbiota composition 28 days later under a chow diet, we could successfully predict the donor testosterone and progesterone levels from the recipient’s mice microbiota by O-PLS modeling (Supplementary Figure 4e, f). After partial Spearman correlation adjusted for donor’s age and obesity status, several bacterial families predicting testosterone levels were the same in mice and humans (Fig. 4f). Specifically, *Sphingobacteriaceae*, *Fibrobacteriaceae*, *Prevotellaceae*, *Cytophagaceae, Idiomarinaceae*, *Flavobacteriaceae*, *Eggerthellaceae*, *Ruminococcaceae*, *Marinifilaceae*, *Deltaproteobacteria*, and *Sporomusaceae* were positively associated with testosterone in both mice and humans, whereas Verrucomicrobia and *Enterobacteriaceae* had negative associations. *Akkermansiaceae* was also negatively associated with human donor testosterone in mice and non-obese subjects.

## Discussion

Sexual dimorphism has long been associated with health and disease [20]. For example, men are at higher risk of CVD than premenopausal women, but this cardioprotection is lost after menopause [21], suggesting the contribution of gonadal steroids on susceptibility to disease. More recently, the gut microbiota has also been implicated on the etiology of several diseases [22–24]. Therefore, the gut microbiota may be the driving force underlying the observed sexual dimorphism in disease susceptibility.

There is some evidence to support that the gut microbiota differs between sex, although the results are inconsistent [1–10, 12, 15], partly because sexual dimorphism in the gut microbiome may also be influenced by diet, age, obesity, ethnicity, and genotype [6]. The findings of the current study confirm some of the previous observations, with higher abundances of *Prevotella* and *Fusobacterium* species in males, and of *Alistipes*, and *Lachnospiraceae* bacterium species in females. In addition, non-obese women also had higher abundances of several *Akkermansia* species compared to non-obese men. Importantly, the majority of the studies did not consider menopausal status as a potential confounding factor. Interestingly, we found that the number of species from genera differentiating men and women was lower after menopause. We also found that premenopausal women had higher abundances of several *Alistipes*, *Bifidobacterium*, and *Ruminococcus* species but lower abundances of *Bacteroides*, *Prevotella*, and *Haemophilus* species compared to postmenopausal women and men. Therefore, our results suggest a masculinization of the gut microbiota composition after menopause.

This androgenization of the microbiome was further corroborated by functional analyses. Therefore, while we found several differentially abundant bacterial pathways between premenopausal women and men, only the arginine biosynthesis and metabolism pathway differed between the postmenopausal and men microbiome. This pathway was enriched in women independently of menopausal status compared to men. It is one of the central pathways for the biosynthesis of arginine, a precursor of nitric oxide (NO), which plays a key role in the regulation of blood pressure. In fact, Forte et al*.* showed that the whole-body production of NO is higher in premenopausal women than in men, although these authors could not decipher the cellular origin of NO [25]. This could partly explain the substantial sex differences observed in hypertension from epidemiological studies [23]. Consistently, alterations in the arginine and proline metabolism have been reported in individuals at high risk of CVD [26] and spontaneously hypertensive rats [27]. We also found a consistent enrichment in the one-carbon pool by folate pathway in the microbiome of pre-menopausal women compared to post-menopausal women and men. Folic acid is the most important determinant of homocysteine levels, whose levels have been related to CVD [28]. Notably, men and post-menopausal women have higher homocysteine levels and are at higher risk of CVD compared to pre-menopausal women [29, 30]. Interestingly, we found that pre-menopausal women had higher abundances of *Lactobacillus plantarum* compared to post-menopausal women and men. Unlike other *Lactobacillus* species, *L. plantarum* is unique in its capacity to synthesize folate [31].

On the other hand, a limited number of studies have shown that the sex difference in the gut microbiota is influenced by the grade of obesity [12, 16]. Accordingly, we found significant differences in the gut microbiota functionality between non-obese premenopausal women and men, but no differences were observed in obese subjects. In addition, none of these studies have taken into account the menopausal status. Similarly, we found that the functional differences in the gut microbiota between pre- and post-menopausal women disappeared in obese women. Therefore, obesity has the effect of erasing the sexual dimorphism associated with the microbiome. Low levels of several plasma steroids, including testosterone and 17-hydroxyprogesterone, have been associated with higher adiposity and visceral fat [32]. These two steroid hormones were precisely among those that did differ between non-obese pre- and post-menopausal women, but not between obese individuals. Due to the relationship between gonadal steroids and the gut microbiome, it is possible that a decreased production of steroid hormones in obese subjects partly explains the lack of sexual dimorphism in the gut microbiota of these individuals.

### Associations of the gut microbiota with gonadal steroids

There is evidence that some bacterial species are able to metabolize and catabolize estrogen and androgens and their precursors, thereby affecting their systemic levels [33–35]. For example, bacteria possess several enzymes involved in the steroid biosynthesis and metabolism such as hydroxysteroid dehydrogenase (HSD). Notably, we found that the gut microbiota of pre-menopausal women was more enriched in steroid biosynthesis and degradation pathways and these microbial pathways had significant associations with the plasmatic levels of testosterone and progesterone. HSD is abundant in species of the Actinobacteria phylum, specifically in *Bifidobacterium* species [36], and Proteobacteria and Firmicutes phyla [37]. Interestingly, we found that premenopausal women had higher abundances of Actinobacteria species, and in particular several *Bifidobacterium* species, compared to men. In addition, we found that several families from the Actinobacteria, Proteobacteria, and Firmicutes phyla were predictive of lower testosterone circulating concentrations.

In line with the ability of the gut microbiota to modulate steroid levels, animal models demonstrate that the microbiome is essential in maintaining regular estrogen cycles, testosterone levels, and reproductive roles in both sexes. Germ-free non-obese diabetic male mice had lower testosterone concentrations than conventional mice [38]. In addition, removal of the microbiota increased the circulating testosterone concentration in female mice but decreased the levels in male mice [38]. The negative associations between the relative abundance of some bacterial species and serum testosterone levels are in line with its removal leading to increased circulating testosterone in females.

In turn, the microbiome may be affected by hormone levels. Previous investigations have focused on the influence of estrogen on microbiota composition [39–45]. For example, the relative abundance of Bacteroidetes was significantly increased in an ovariectomized rat model [46]. In another study, normal females had significantly lower Proteobacteria abundance and Firmicutes to Bacteroidetes ratio, but higher *Bifidobacterium* to *Enterobacteriaceae* ratio and increased *Akkermansia* abundance, compared to normal male and ovariectomized (OVX) females [45]. Some of these observations were recapitulated with 17-β-estradiol treatments in males and OVX females. Treatment with progesterone in OVX mice resulted in a significant change in the gut microbiota composition, with particular increases in *Lactobacillus* species [47]. In women, the relative abundance of the *Gammaproteobacteria* class and an unknown family from *Myxococcales* correlated positively with estradiol levels, while *Prevotellaceae* had a negative correlation [13]. Consistently, we found that *Prevotellaceae* family was positively associated with testosterone levels, whereas *Gammaproteobacteria*, *Lactobacillaceae*, and *Myxococcaceae* had negative associations.

Post-menopausal women did not exhibit higher levels of testosterone than pre-menopausal women suggesting that a mechanism other than androgenization may be also responsible for the differences in gut microbiota between pre-menopausal women vs. post-menopausal women and men.

### Effects of testosterone

Only a few studies reported the associations between the gut microbiota and testosterone. Prenatal exposure to testosterone cypionate in female rats resulted in a lower abundance of *Akkermansia*, *Bacteroides*, *Lactobacillus*, and *Clostridium* in adult female offspring [48]. Gonadectomy along with a high fat diet was associated with increased genera of *Ruminococcaceae* family in male mice of three different strains and reduced *Akkermansia* genus in female mice [49]. However, males on a chow diet had higher *Ruminococacceae* than females. In a recent investigation using 16S rRNA sequencing methods, the authors observed significant positive correlations between the relative abundance of bacteria in the *Actinobacter*, *Dorea*, *Ruminococcus*, and *Megamonas* genera and serum testosterone concentrations in men [50]. An indirect way to consider the effects of testosterone levels is through women or animal models of polycystic ovary syndrome (PCOS), which is characterized by an excessive production of androgens or testosterone. In female rats, the increase in testosterone after Letrozole-induced PCOS led to decreased *Lactobacillus*, *Ruminococcus*, and *Clostridium*, but higher *Prevotella* [51]. Letrozole treatment of adult mice also resulted in a higher relative abundance of genera from *Lachnospiraceae*, *Ruminococcaceae*, and *Peptococcaceae*, and lower *Lactobacillus* [52]. In a recent human study, Bacteroidetes, Firmicutes, and Verrucomicrobia phyla differed between women with PCOS and controls, with obesity having a driving role in the development of dysbiotic microbiota [53]. In particular, women with PCOS had higher relative abundances of several *Ruminococcus* spp. In line with these results, in the current study, the abundance of some *Lachnospiraceae bacterium* (Firmicutes) and *Ruminococcus* spp. (Firmicutes) was higher in males vs. pre-menopausal females, whereas several *Prevotella* spp. (Bacteroidetes) were higher in males compared to women. We also found that non-obese women also had higher abundances of several *Akkermansia* spp*.* (Verrucomicrobia) compared to non-obese men. O-PLS modeling also revealed a negative association between Verrucomicrobia and *Akkermansiaceae* family and testosterone levels in non-obese individuals. However, the most consistent association with testosterone in O-PLS models were with *Prevotellaceae*, *Cytophagaceae*, *Ruminococcaceae*, *Fibrobacteriaceae*, Sphingobacteriaceae, and *Idiomarinaceae*. Remarkably, this microbial signature predictive of the testosterone levels could be transmitted to mice by faecal microbiota transplantation (FMT), being this signature in mice able to predict donor’s serum testosterone 28 days later. However, we must point out the one limitation of the present FMT study is that only male mice were used.

Although the number was small, women with PCOS did not have elevated testosterone levels when compared with the rest of premenopausal women and did not significantly influence the findings concerning testosterone levels. The median testosterone levels in women with PCOS were 0.0785 [0.0531-0.1165], in the range of that observed among premenopausal women and with no outliers. These women with PCOS had also low androstenedione levels compared to the other pre-menopausal women (0.1057, 0.1431, and 0.3008 in the range from 0.069-1.35 observed for androstenedione in pre-menopausal women). Similar results were observed for DHEA and DHT. In addition, we performed the KEGG metagenome functional analyses again removing these three pre-menopausal women and the results were almost the same.

## Conclusions

In conclusion, our results evidence a clear difference in the gut microbial composition and functionality between men and women, which is influenced by both menopausal and obesity status. Therefore, menopause is proposed to induce an androgenization of the microbiome, whereas obesity overrides the sex and menopause differences observed in non-obese individuals. The gut microbiota composition was tightly linked to the circulating levels of gonadal steroids, particularly testosterone.

## Methods

### Clinical study (cross-sectional)

#### Recruitment of study subjects

From January 2016 to October 2017, a cross-sectional case-control study was undertaken in the Endocrinology Department of JosepTrueta University Hospital. We included consecutive subjects with obesity (body mass index, BMI ≥ 30 kg/m^2^) and age- and sex-matched nonobese subjects (18.5 < BMI < 30 kg/m^2^), with an age range of 27.2-66.6 years (*n* = 131). Exclusion criteria were the following: type 2 diabetes mellitus, chronic inflammatory systemic diseases, acute or chronic infections in the previous month; use of antibiotic, antifungal, antiviral, or treatment with proton-pump inhibitors; severe disorders of eating behavior or major psychiatric antecedents; and excessive alcohol intake (≥ 40 g OH/day in women or 80 g OH/day in men). The institutional review board—Ethics Committee and the Committee for Clinical Research (CEIC) of Dr.JosepTrueta University Hospital (Girona, Spain) approved the study protocol, and informed written consent was obtained from all participants.

#### Clinical and laboratory parameters

Body composition was assessed using a dual energy X-ray absorptiometry (DEXA, GE Lunar, Madison, Wisconsin).

### Longitudinal cohort

A total of *n =* 81 subjects were followed for 1 year and plasma samples were collected after 1 year of follow-up for the determination of steroids.

### Extraction of fecal genomic DNA and whole-genome shotgun sequencing

Total DNA was extracted from frozen human stools using the QIAamp DNA mini stool kit (Qiagen, Courtaboeuf, France). Quantification of DNA was performed with a Qubit 3.0 fluorometer using the QubitTM dsDNA HS assay kit (Thermo Fisher Scientific, Carlsbd, CA, USA), and 1 ng of each sample (0.2 ng/μl) was used for shotgun library preparation for high-throughput sequencing, using the Nextera DNA Flex Library Prep kit (Illumina, Inc., San Diego, CA, USA) according to the manufacturers’ protocol. Sequencing was carried out on a NextSeq 500 sequencing system (Illumina) with 2 × 150-bp paired-end chemistry (120Gb/120 millions of reads), at the facilities of the Sequencing and Bioinformatic Service of the FISABIO (Valencia, Spain).

Metagenome sequences were trimmed and quality controlled using the PRINSEQ-lite-0.20.4 program [54], and forward and reverse reads were joined using FLASH-1.2.11 [55], applying a minimum overlap of 10, maximum overlap of 150, and maximum mismatch density of 0.1. Host reads were removed by mapping against the host reference genome, by using bowtie2-2.3.4.3 [56] with end-to-end and very sensitive options. For functional analyses an assembly of the host-free reads into contigs was implemented by MEGAHIT v1.1.2 [57], followed by a mapping of those reads against the contigs with bowtie2, appending the reads that did not assemble to the contigs. Next, the program Prodigal v2.6.3 [58] was used for predicting open reading frames (orfs). The functional annotation was carried out with HMMER [59] against the Kyoto Encyclopedia of Genes and Genomes (KEGG) database [60] to obtain the functional at three levels, subcategory, pathway, and annotation of the genes. The filtering of the best annotations and the assignment of the orf annotation to every read were carried out using the statistical package R 3.6.0 [61], which also was used to count the aligned reads and to add the category and its coverage, and finally to build abundance matrices.

In parallel, taxonomic annotation was implemented with Kaiju v1.6.2 [62] on the concatenated host-free reads, and the resulting file was split by sample. Addition of lineage information, lineage parsing for undetermined ranks, counting of taxa, and generation of the operational taxonomic unit (OTU) absolute and relative abundance matrices for all samples were performed using the package R.

### Full automated method for determination of steroids

The determination of steroids in serum was carried out by liquid chromatography with tandem mass spectrometry (LC–MS/MS) detection. Sample preparation involved solid-phase extraction (SPE) in an automated unit from Spark Holland (Emmen, The Netherlands), which was on-line coupled to a LC–MS/MS with a triple quadrupole mass detector from Agilent (Palo Alto, USA). A volume of 175 μL of serum is introduced in an opaque vial with a glass insert and located in the autosampler of the SPE system. The online SPE protocol consists of a first equilibration step of the sorbent cartridge (C18 HySphere, Spark-Holland, 10 mm length and 2 mm diameter, 8 μm particle size), with 10 mL of methanol (MeOH) followed by two solvation steps with 1 mL of 10% (v/v) acetonitrile (ACN) in water containing 0.1% (v/v) of formic acid. Meanwhile, the sample loop is filled with 100 μL of sample with a subsequent load in the sorbent cartridge with 2 mL of 10% (v/v) ACN in water containing 0.1% (v/v) of formic acid. The loading step leads to the consequent retention of the analytes in the sorbent and the removal of sample matrix components to waste. Then, the sorbent is rinsed with 1 mL of 20% (v/v) ACN in water and, then, the analytes are eluted with the chromatographic mobile phase (for 9 min and 30 s elution time).

Chromatographic separation of steroids was carried out by a Kinetex C18 analytical column (particle size 2.6 μm, 10 cm length, and 3 mm inner diameter) from Phenomenex (Torrance, CA, USA). A security guard C18 cartridge (4 mm length and 3 mm inner diameter) from Phenomenex was used to preserve the integrity of the analytical column. The initial chromatographic mobile phase was composed of 40% (v/v) MeOH in water containing 0.2 mM NH4F as an ionizing agent. A first gradient was applied for 3.5 min up to 68% of MeOH, a second gradient for 6 min up to 71% of MeOH, followed by a third gradient for 4 min to 80% of MeOH and, finally, a last gradient for 1 min up to 100% of MeOH. The temperature of the chromatographic column was set at 35 °C and the flow rate is 300 μL min-1.

The MS/MS detection was carried out in multiple reaction monitoring (MRM) with electrospray ionization (ESI) in fast switching polarity mode. The capillary voltage was set at +/−3.5 kV and the nozzle voltage at 0.5 kV. The nebulizer pressure was set at 45 psi and the flow of N2 as drying gas at 12 L min-1 and 325 °C. Parameters for MRM detection are listed in Supplementary Table 5. Calibration models were prepared by analysis of aliquots of a serum pool spiked with variable concentrations of the target steroids. Endogenous content of each steroid in the sample loop was subtracted in the preparation of the calibration models. Isotopically labeled steroids were used as internal standards for quantitative analysis of structurally similar analytes.

### Animal procedures

Eight-weeks-old male C57BL/6 J mice (Charles River, France), weighing 20–26 g at the beginning of the experiment were used in this study. Mice were housed individually in controlled laboratory conditions with the temperature maintained at 21 ± 1 °C and humidity at 55 ± 10% during all the study. Animal procedures were conducted in strict accordance with the guidelines of the European Communities Directive 86/609/EEC regulating animal research and were approved by the local ethical committee (CEEA-PRBB). All the experiments were performed under blinded conditions.

Then, mice were given a cocktail of ampicillin and metronidazole, vancomycin (all at 500 mg/L), ciprofloxacin HCl (200 mg/L), imipenem (250 mg/L) once daily for 14 consecutive days in drinking water, as previously described [63]. Seventy-two hours later, animals were colonized via daily oral gavage of donor microbiota (150 μL) for 3 days. Animals were orally gavaged with fecal material from age- and obesity-matched healthy volunteers (*n* = 22, 11 men and 11 women). To offset potential confounder and/or cage effects and to reinforce the donor microbiota phenotype, booster inoculations were given twice per week throughout the study. Animals were maintained on normal mouse chow diet (rat and mouse no. 1 maintenance diet, Special Diet Services®) and water ad libitum, and were weighed every week. Food intake was the same in the two groups. At the end of the study, the animals were consecutively sacrificed. The cecum was removed, weighed and stored, and the feces collected and stored at −80 °C for further microbiota analysis. Fecal microbiota composition from mice was analyzed following the same procedures as for humans.

### Statistical analysis

Normal distribution and homogeneity of variances in clinical variables were tested using the Shapiro-Wilk and Levene’s tests. Results are expressed as number and frequencies for categorical variables, mean and standard deviation for normal distributed continuous variables, and median and interquartile range for non-normal distributed continuous variables. Differences between study groups were assessed using the *χ*^2^ for categorical variables, one-way ANOVA for normal quantitative variables, and Kruskal-Wallis tests for non-normal quantitative variables. These analyses were performed using SPSS version 19 (SPSS, Inc, Chicago, IL).

For metagenomics data, taxa were first filtered so that only those with more than 10 reads in at least two samples were selected. Then, differential bacterial taxa or KEGG pathways between groups were identified with the R package DESeq2 [64]. It uses a generalized linear model of counts based on a negative binomial distribution, scaled by a normalization factor that accounts for differences in sequencing depth between samples. Significance testing was then assessed using the Wald test and the *p* values corrected for multiple comparisons by the Benjamini-Hochberg procedure (*p*FDR) [65]. In all models, age and obesity status were included as covariates.

Predications of circulating gonadal steroid levels from bacterial families were performed using orthogonal partial least squares (O-PLS) models with unit variance using in-house MATLAB scripts. The abundances of bacterial families after applying a variance stabilizing transformation using DESeq2 were used as the descriptor matrices (X) to predict the testosterone levels as the response (Y). The predictive performance (*Q*^2^*Y*) of each model was calculated using a tenfold cross-validation approach, and model validity was established by permutation testing (1000 permutations). The significance of O-PLS correlation coefficients was adjusted by the Benjamini-Hochberg method (*p*FDR).

## Supplementary information

**Additional file 1: Supplementary Table 1.** Clinical characteristics of subjects after 1-year follow-up according to the gender and menopausal status.

**Additional file 2: Supplementary Table 2.** DESeq2 results for the differential expressed bacterial taxa between pre-menopausal women and men.

**Additional file 3: Supplementary Table 3.** DESeq2 results for the differential expressed bacterial taxa between post-menopausal women and men.

**Additional file 4: Supplementary Table 4.** DESeq2 results for the differential expressed bacterial taxa between pre-menopausal women and post-menopausal women.

**Additional file 5: Supplementary Table 5.** MRM parameters for determination of steroids and isotopically labelled standards by LC–MS/MS.

**Additional file 6: Supplementary Figure 1.** Associations of gut microbiota composition in non-obese and obese subjects and bacterial families with gender and menopause status. Alpha diversity indices in a) non-obese and b) obese individuals. Beta diversity in non-obese subjects measured by c)Bray-Curtis and d) weighted unifrac. Beta diversity in obese subjects measured by e) Bray-Curtis and f) weighted unifrac. Overall differences in the microbiome composition among groups were assessed by PERMANOVA using 1000 permutations and pairwise differences between groups were assessed using the pairwise.adonis function adjusted for Bonferroni correction. *,*P* < 0.05; **, *P* < 0.01.g) Volcano plot of differential bacterial families abundance analysisbetween pre-menopausal women and men, h) post-menopausal women and men, and i) pre- and post-menopausal women, as identified by DESeq2 from shotgun metagenomic sequencing data, adjusting for age and obesity status. For each family, the fold change and the *p* values corrected for multiple comparisons by the Benjamini-Hochberg procedure (*p*FDR) are plotted. Significantly different taxa (FC > 1 and *p*FDR < 0.05) are coloured according to phylum.

**Additional file 7: Supplementary Figure 2.** Associations of gut microbiota functionality with gender and menopause status in non-obese subjects. **a)** Fold change for the significant differential KEGG pathways between pre-menopausal women and men, and **b)** pre- and post-menopausal women, identified by DESeq2 adjusting for age and obesity status. Bars are colored according to the Benjamini-Hochberg corrected *p* values (*p*FDR).

**Additional file 8: Supplementary Figure 3.** Gender and menopausal status differences in gonadal steroids according to the obesity status. Boxplots for the concentrations of progestin,androgens, and estrogens converted to base 10 logarithmic values. Differences among groups were analyzed by a Kruskal-Wallis test, and pair-wise comparisons were assessed by the Wilcoxon test. Significant differences are highlighted in bold italics.

**Additional file 9: Supplementary Figure 4.** Gut microbial associations with circulating testosterone concentrations. **a)** Permutation tests for the goodness-of-fit (*R*^2^*Y*) and goodness of prediction (*Q*^2^*Y*) for the O-PLS model predicting plasma testosterone levels from bacterial families in non-obese individuals. **b)** Significant gut bacterial families identified by O-PLS modeling. **c)** Permutation tests for the goodness-of-fit (*R*^2^*Y*) and goodness of prediction (*Q*^2^*Y*) for the O-PLS model predicting plasma testosterone levels after 1-year follow-up from bacterial families at baseline in humans. **d)** Significant gut bacterial families identified by O-PLS modeling. **e)** Permutation tests for the goodness-of-fit (*R*^2^*Y*) and goodness of prediction (*Q*^2^*Y*) for the O-PLS model predicting human donor circulating testosterone and **f)** progesterone concentrations from recipient’s mice bacterial families.

## Data Availability

The datasets used and/or analyzed during the current study are available from the corresponding author on reasonable request.
