# Supplementary material for: Gut microbiota steroid sexual dimorphism and its impact on gonadal steroids: influences of obesity and menopausal status
Source: Microbiome. 2020 Sep 20;8:136. doi: 10.1186/s40168-020-00913-x (PMC7504665; doi:10.1186/s40168-020-00913-x)
Supplement: Supplementary file 2 — Additional file 1: Supplementary Table 1. Clinical characteristics of subjects after 1-year follow-up according to the gender and menopausal status. [file 40168_2020_913_MOESM1_ESM.docx]

**Table S1.** Clinical characteristics of subjects after 1-year follow-up according to the gender and menopausal status.

| **Clinical data** | **Pre-menopausal**  **(*n*=26)** | **Post-menopausal**  **(*n*=26)** | **Men**  **(*n*=29)** | ***P*** |
| --- | --- | --- | --- | --- |
| Age | 41.8 [34.9-48.0] | 59.5 [55.3-61.0] | 49.2 [43.2-60.0] | <0.001 |
| Policystic Ovary Syndrome (%) | 0 (0%) | 0 (0%) | 0 (0%) | - |
| Alcohol intake (g/day) | 0.0 [0.0-1.6] | 0.72 [0.0-1.85] | 2.3 [0.0-12.9] | 0.018 |
| Smoking (no, former, yes) (%) | 61.5, 26.9, 11.5 | 26.9, 65.4, 7.7 | 44.4, 42.0, 13.6 | 0.033 |
| Obesity (%) | 53.8 | 30.8 | 55.2 | 0.134 |
| BMI (kg/m^2^) | 31.1 [24.0-42.1] | 26.0 [23.5-31.0] | 31.9 [27.4-39.8] | 0.108 |
| Fat mass (kg) | 30.4 [21.2-50.0] | 24.2 [21.4-33.9] | 27.3 [21.6-33.9] | 0.838 |
| SBP (mmHg) | 125.6 (16.9) | 129.9 (12.9) | 129.2 (12.7) | 0.569 |
| DBP (mmHg) | 72.8 (13.6) | 74.3 (9.80) | 75.6 (8.02) | 0.519 |
| HDL cholesterol (mg/dL) | 57.0 [48.5-64.3] | 64.5 [54.0-79.8] | 49.0 [42.0-60.0] | 0.001 |
| Triglycerides (mg/dL) | 73.5 [63.3-96.0] | 93.0 [56.4-117.0] | 107 [78.0-151.0] | 0.034 |
| Fasting plasma glucose (mg/dl) | 93.5 [88.5-99.5] | 95.5 [90.0-102.5] | 96.0 [92.5-103.5] | 0.121 |
| IVGTT (80-120 min) (mg/dL) | 99.4 (8.90) | 100.6 (7.21) | 104.8 (11.2) | 0.259 |
| M-clamp (mg/(kg·min)) | 6.33 [4.0-10.5] | 8.90 [7.49-11.1] | 4.75 [2.34-8.04] | 0.045 |
| HbA1c (%) | 5.40 (0.32) | 5.50 (0.36) | 5.57 (0.27) | 0.057 |
| hs-CRP (mg/dL) | 1.95 [0.64-3.86] | 1.61 [0.67-4.91] | 1.91 [1.26-3.10] | 0.763 |
|  |  |  |  |  |
