# Supplementary material for: Gut microbiota steroid sexual dimorphism and its impact on gonadal steroids: influences of obesity and menopausal status
Source: Microbiome. 2020 Sep 20;8:136. doi: 10.1186/s40168-020-00913-x (PMC7504665; doi:10.1186/s40168-020-00913-x)
Supplement: Supplementary file 6 — Additional file 5: Supplementary Table 5. MRM parameters for determination of steroids and isotopically labelled standards by LC–MS/MS. [file 40168_2020_913_MOESM5_ESM.docx]

**Supplementary Table 4.** MRM parameters for determination of steroids and isotopically labelled standards by LC–MS/MS.

| **Analyte** | **Neutral mass** | **Polarity** | **Transition (*m/z)*** | **Q1 voltage (V)** | **CE (eV)** |
| --- | --- | --- | --- | --- | --- |
| **17-OHP_5_** | 332.5 | **–** | 331 -> 287 | 55 | 16 |
| **17-OHP_4_** | 330.5 | – | 329 -> 285 | 70 | 18 |
| **P_5_** | 316.5 | + | 299 -> 281 | 80 | 20 |
| **P_5_-d_4_** | 320.5 | + | 303 -> 285 | 90 | 5 |
| **P_4_** | 314.5 | + | 315 -> 97 | 85 | 20 |
| **P_4_-C_3_** | 317.5 | + | 318 -> 100 | 70 | 20 |
| **A_4_** | 286.4 | + | 287 -> 97 | 80 | 20 |
| **DHEA** | 288.4 | + | 271 -> 253 | 70 | 7 |
| **DHEA-d_6_** | 294.4 | + | 277 -> 259 | 80 | 7 |
| **DHT** | 290.4 | + | 291 -> 255 | 80 | 11 |
| **T** | 288.4 | + | 289 -> 97 | 100 | 18 |
| **E_1_** | 270.4 | – | 269 -> 145 | 140 | 36 |
| **E_1_-C_3_** | 273.4 | – | 272 -> 148 | 145 | 36 |
| **E_2_** | 272.4 | – | 271 -> 145 | 135 | 40 |
| **E_2_-C_3_** | 275.4 | – | 274 -> 148 | 150 | 40 |
| **21-OHP_4_** | 330.5 | + | 331 -> 97 | 75 | 20 |
| **F** | 362.4 | + | 363 -> 121 | 70 | 22 |
| **E** | 360.4 | – | 327 -> 312 | 70 | 16 |
| **B** | 346.4 | – | 345 -> 241 | 60 | 17 |
| **S** | 346.5 | – | 313 -> 285 | 80 | 24 |
| **S-d_5_** | 351.5 | – | 318 -> 290 | 60 | 24 |
| **A** | 360.4 | – | 359 -> 189 | 70 | 15 |
