# Supplementary figures and images for: Gut microbiota steroid sexual dimorphism and its impact on gonadal steroids: influences of obesity and menopausal status
Source: Microbiome. 2020 Sep 20;8:136. doi: 10.1186/s40168-020-00913-x (PMC7504665; doi:10.1186/s40168-020-00913-x)

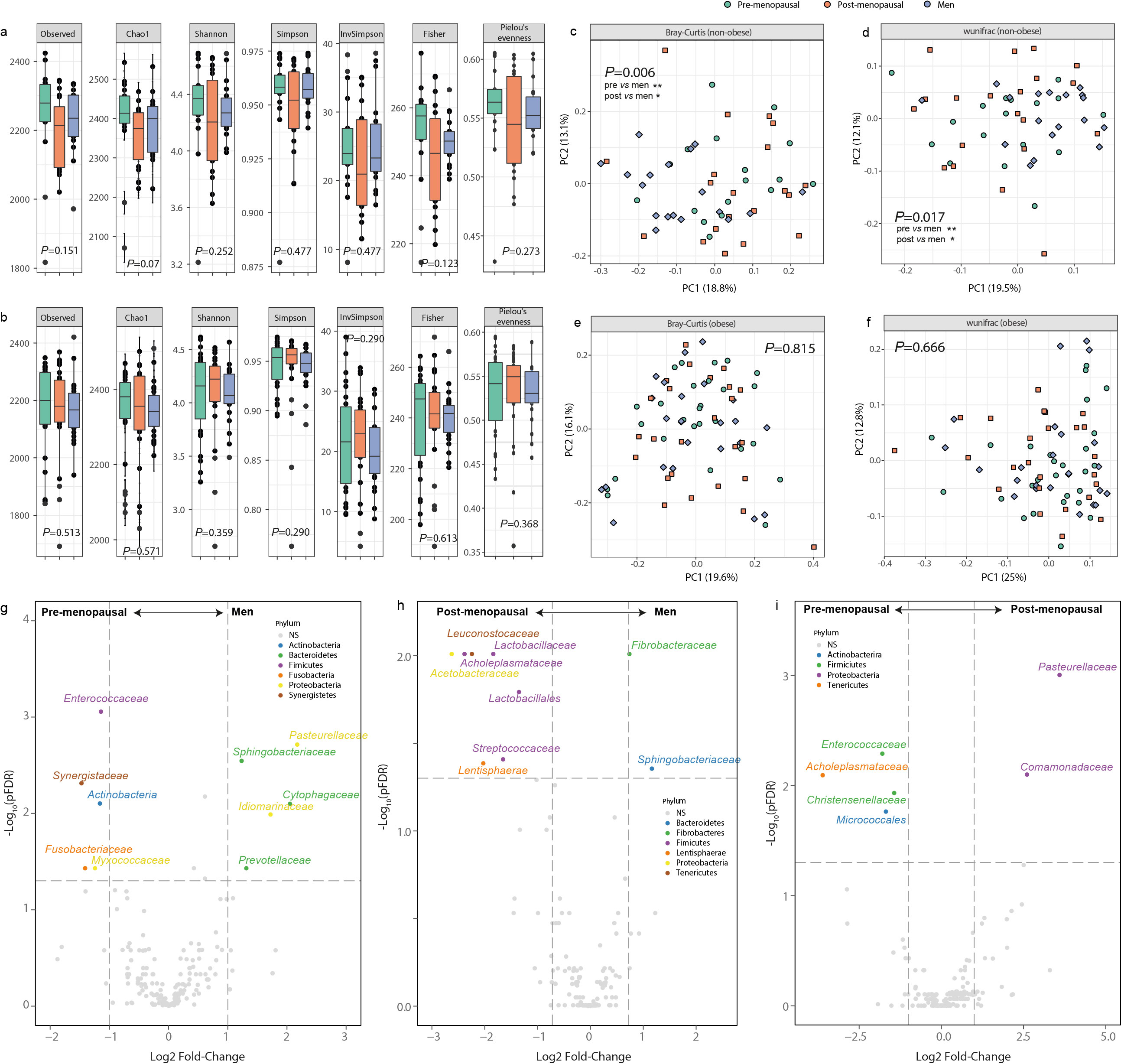

Supplement: Supplementary file 7 — Additional file 6: Supplementary Figure 1. Associations of gut microbiota composition in non-obese and obese subjects and bacterial families with gender and menopause status. Alpha diversity indices in a) non-obese and b) obese individuals. Beta diversity in non-obese subjects measured by c)Bray-Curtis and d) weighted unifrac. Beta diversity in obese subjects measured by e) Bray-Curtis and f) weighted unifrac. Overall differences in the microbiome composition among groups were assessed by PERMANOVA using 1000 permutations and pairwise differences between groups were assessed using the pairwise.adonis function adjusted for Bonferroni correction. *,P < 0.05; **, P < 0.01.g) Volcano plot of differential bacterial families abundance analysisbetween pre-menopausal women and men, h) post-menopausal women and men, and i) pre- and post-menopausal women, as identified by DESeq2 from shotgun metagenomic sequencing data, adjusting for age and obesity status. For each family, the fold change and the p values corrected for multiple comparisons by the Benjamini-Hochberg procedure (pFDR) are plotted. Significantly different taxa (FC > 1 and pFDR < 0.05) are coloured according to phylum. [file 40168_2020_913_MOESM6_ESM.jpg]

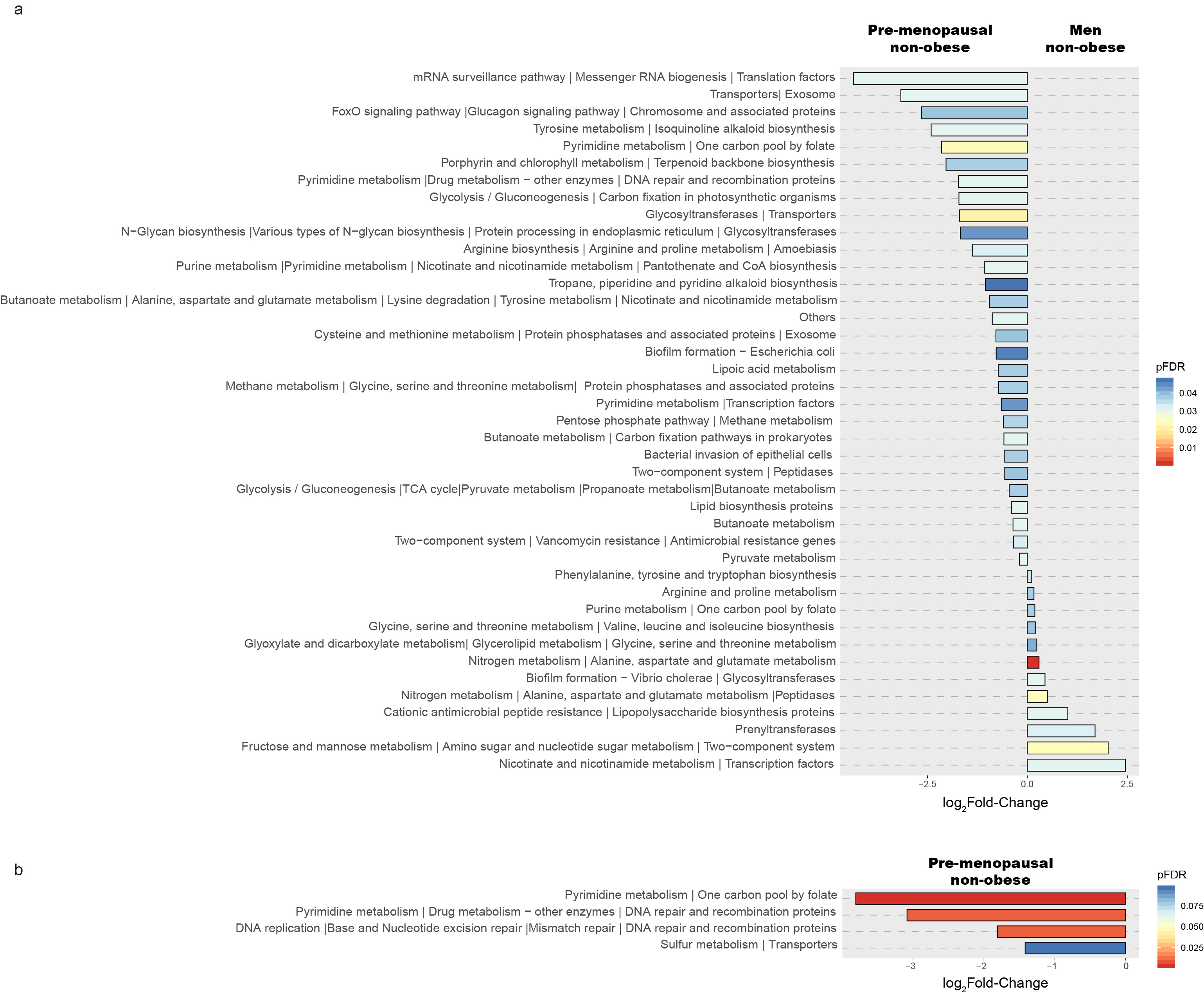

Supplement: Supplementary file 8 — Additional file 7: Supplementary Figure 2. Associations of gut microbiota functionality with gender and menopause status in non-obese subjects. a) Fold change for the significant differential KEGG pathways between pre-menopausal women and men, and b) pre- and post-menopausal women, identified by DESeq2 adjusting for age and obesity status. Bars are colored according to the Benjamini-Hochberg corrected p values (pFDR). [file 40168_2020_913_MOESM7_ESM.jpg]

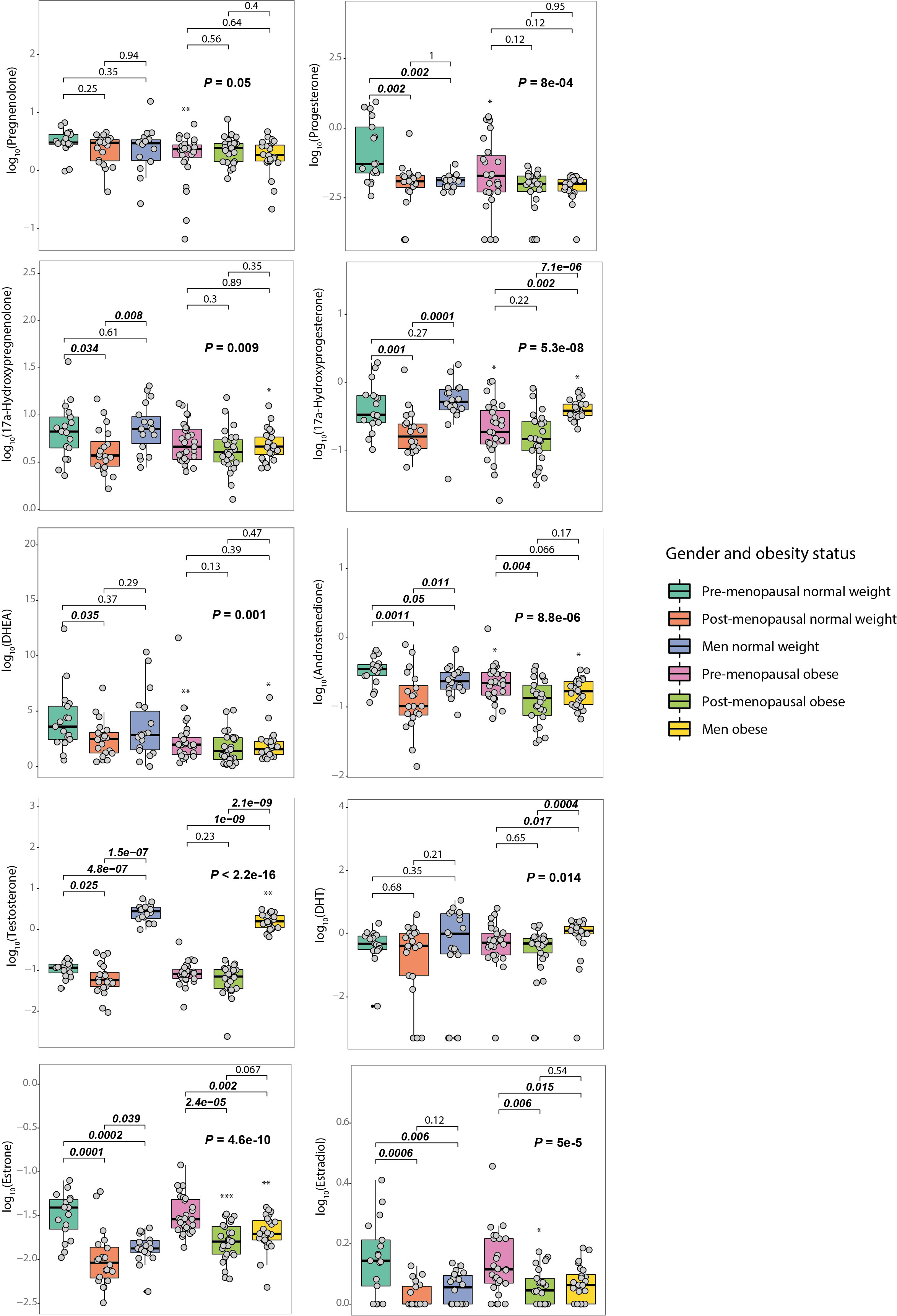

Supplement: Supplementary file 9 — Additional file 8: Supplementary Figure 3. Gender and menopausal status differences in gonadal steroids according to the obesity status. Boxplots for the concentrations of progestin,androgens, and estrogens converted to base 10 logarithmic values. Differences among groups were analyzed by a Kruskal-Wallis test, and pair-wise comparisons were assessed by the Wilcoxon test. Significant differences are highlighted in bold italics. [file 40168_2020_913_MOESM8_ESM.jpg]

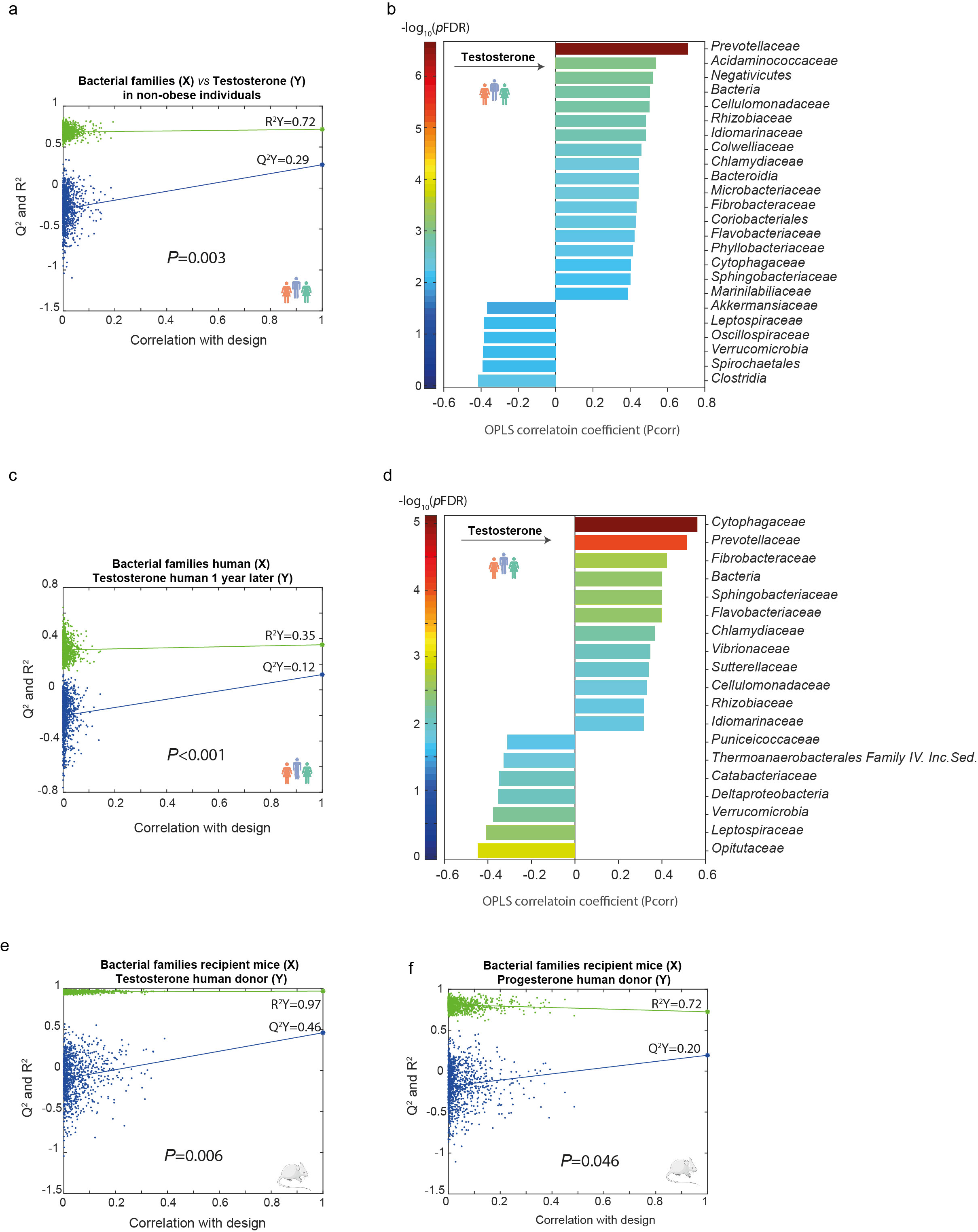

Supplement: Supplementary file 10 — Additional file 9: Supplementary Figure 4. Gut microbial associations with circulating testosterone concentrations. a) Permutation tests for the goodness-of-fit (R2Y) and goodness of prediction (Q2Y) for the O-PLS model predicting plasma testosterone levels from bacterial families in non-obese individuals. b) Significant gut bacterial families identified by O-PLS modeling. c) Permutation tests for the goodness-of-fit (R2Y) and goodness of prediction (Q2Y) for the O-PLS model predicting plasma testosterone levels after 1-year follow-up from bacterial families at baseline in humans. d) Significant gut bacterial families identified by O-PLS modeling. e) Permutation tests for the goodness-of-fit (R2Y) and goodness of prediction (Q2Y) for the O-PLS model predicting human donor circulating testosterone and f) progesterone concentrations from recipient’s mice bacterial families. [file 40168_2020_913_MOESM9_ESM.jpg]
